# HIV-1 Integrase-Targeted Short Peptides Derived from a Viral Protein R Sequence

**DOI:** 10.3390/molecules23081858

**Published:** 2018-07-26

**Authors:** Xue Zhi Zhao, Mathieu Métifiot, Evgeny Kiselev, Jacques J. Kessl, Kasthuraiah Maddali, Christophe Marchand, Mamuka Kvaratskhelia, Yves Pommier, Terrence R. Burke

**Affiliations:** 1 Chemical Biology Laboratory, Center of Cancer Research, Frederick, MD 21702, USA; 2 Developmental Therapeutics Branch and Laboratory of Molecular Pharmacology, Center for Cancer Research, National Cancer Institute, National Institutes of Health, Bethesda, MD 20892, USA; mathieu.metifiot@u-bordeaux.fr (M.M.); evgeny.kiselev@nih.gov (E.K.); maddalik@makorelabs.com (K.M.); marchanc@mail.nih.gov (C.M.); Yves.Pommier@nih.gov (Y.P.); 3 College of Pharmacy and Center for Retrovirus Research, The Ohio State University, Columbus, OH 43210, USA; Jacques.Kessl@usm.edu (J.J.K.); MAMUKA.KVARATSKHELIA@UCDENVER.EDU (M.K.); 4 Department of Chemistry and Biochemistry, The University of Southern Mississippi, Hattiesburg, MS 39406, USA; 5 Division of Infectious Diseases, University of Colorado School of Medicine, Aurora, CO 80045, USA

**Keywords:** HIV-1 integrase, viral protein R, photoaffinity probe, inhibitor

## Abstract

HIV-1 integrase (IN) inhibitors represent a new class of highly effective anti-AIDS therapeutics. Current FDA-approved IN strand transfer inhibitors (INSTIs) share a common mechanism of action that involves chelation of catalytic divalent metal ions. However, the emergence of IN mutants having reduced sensitivity to these inhibitors underlies efforts to derive agents that antagonize IN function by alternate mechanisms. Integrase along with the 96-residue multifunctional accessory protein, viral protein R (Vpr), are both components of the HIV-1 pre-integration complex (PIC). Coordinated interactions within the PIC are important for viral replication. Herein, we report a 7-mer peptide based on the shortened Vpr (69–75) sequence containing a biotin group and a photo-reactive benzoylphenylalanyl residue, and which exhibits low micromolar IN inhibitory potency. Photo-crosslinking experiments have indicated that the peptide directly binds IN. The peptide does not interfere with IN-DNA interactions or induce higher-order, aberrant IN multimerization, suggesting a mode of action for the peptide that is distinct from clinically used INSTIs and developmental allosteric IN inhibitors. This compact Vpr-derived peptide may serve as a valuable pharmacological tool to identify a potential new pharmacologic site.

## 1. Introduction

HIV-1 integrase (IN) is a virally encoded polynucleotidyl transferase that inserts reverse-transcribed viral cDNA into the host genome using two sequential reactions, cleavage of the 3′-dinucleotides from viral DNA, referred to as 3′-processing (3′-P), and insertion of the processed ends of viral DNA into the host genome, termed strand transfer (ST) [1]. Integrase strand transfer inhibitors (INSTIs) are the newest class of AIDS therapeutic, with three FDA-approved agents now being on the market (raltegravir (RAL, October 2007) [2], elvitegravir (EVG, August 2012) [3] and dolutegravir (DTG, August 2013) [4]). Drug resistance can be an unwanted side effect of long-term therapy using anti-AIDS drugs and the most recently introduced INSTIs do not present an exception [5,6]. Therefore, efforts continue to develop new inhibitors that are not affected by the extant INSTI-resistant forms of IN. 

Binding of INSTIs to the enzyme catalytic site occurs in the cytoplasm, where IN exists in multimeric form as part of a pre-integration complex (PIC), which contains other viral proteins that include reverse transcriptase (RT), matrix protein (MA) and viral protein R (Vpr). Multiple host cofactors are also involved in the formation of the PIC, such as lens epithelium-derived growth factor (LEDGF)/transcriptional coactivator p75. Full proviral DNA integration requires entry of the PIC into the nucleus and proper chromosomal insertion. The catalytic activity and nuclear localization of IN are facilitated by cellular and viral proteins including Vpr and LEDGF [7,8]. Synthetic peptides that mimic structural elements employed by these cofactors to carry out their roles in viral replication may provide new classes of inhibitors that are less affected by resistance mutants arising from catalytic site-binding INSTIs [9,10,11]. Indeed, peptides based on sequences of the LEDGF integrase-binding domain (IBD) have been shown to inhibit catalytic function, in part by shifting the equilibrium of IN oligomerization [12]. As Vpr exerts its complex and not completely understood role in the pathogensis of HIV-1 in non-dividing cells, it potentially affords a new and alternate non-catalytic target for therapeutic exploration [13,14,15,16,17,18].

**Figure 1:**
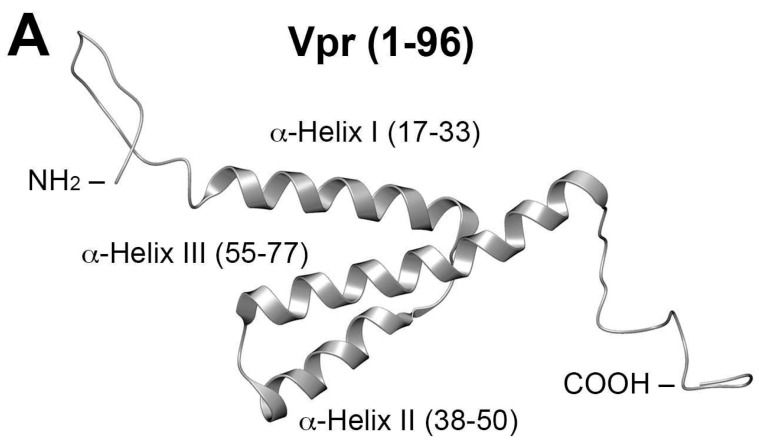

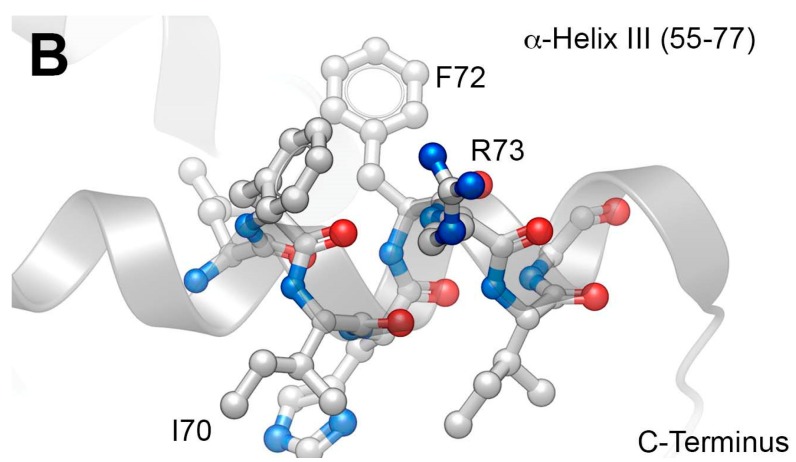
NMR solution structure of Vpr (1–96) (PDB accession code: 1M8L [19]). (**A**) Full-length structure showing helices; (**B**) Closeup of α-helix III showing residues “FIHFRIG” (69–75) overlain on a ribbon depiction.

Vpr is a 96-residue protein having flexible N- and C-termini. These bracket three internal, well-defined α-helices (I–III; residues 17–33, 38–50 and 56–77, respectively), which are folded about a hydrophobic core (Figure 1A) [19]. An early report showed that C-terminal residues [Vpr (52–96)] are responsible for binding of IN to viral DNA, with evidence suggesting that residues 88–96 are particularly important [20,21]. A screen of peptides sharing 11-amino acid overlaps and covering the full Vpr protein, identified Vpr (61–75) (IRILQQLLFIHRIG) in α-helix III as being able to inhibit most potently both the 3′-P and ST in vitro (Figure 1B) [22]. Recent work examining the ability of peptides derived from HIV-1 gene products to inhibit IN has identified the Vpr (64–69) sequence “LQQLLF” as being shared by pooled 15-mer Vpr-derived peptides. These peptides showed good ST-inhibitory potencies (IC_50_ = 68 µM) [23]. Their sequence occupies the central region of the Vpr C-terminal α-helix. Extending the sequence to cover more of the α-helix by adding the Vpr (69–75) residues (IHFRIG) was shown to increase inhibitory potency [23]. In further work, these authors examined the effects on IN inhibitory potencies incurred by employing ring-closing metathesis “stapling” to stabilize α-helix conformations of the Vpr (58–75)-derived sequence, “EAIIRILQQLL FIHFRIG” [24]. These structural modifications left unchanged the C-terminal “FIHFRIG”-containing region.

The assays described above were performed using purified IN in the absence of other proteins that comprise essential components of the PIC. There is no evidence that Vpr binds directly to IN within a cellular context [18]. In light of this, the ability of Vpr-derived peptides to inhibit IN in in vitro assays may reflect binding to sites on IN that may be unrelated to the other functions of Vpr in vivo. Our current work was undertaken to identify minimal Vpr-derived sequences that retain micromolar IN inhibitory potencies in in vitro assays and to introduce photoaffinity crosslinking functionality into the best peptides to provide pharmacological probes that might be useful in delineating regions of IN that are important for peptide binding [25].

## 2. Results and Discussion

### 2.1. Development of a Core Vpr-Derived IN Inhibitory Peptide

We began our work by exploring the Vpr (69–75) peptide “FIHGRIG”. This sequence was of interest, not only because it had been shown to increase inhibitory potency when added to shorter Vpr (65–69) derived peptides from the Vpr α-helix III, but also because it is contained within a Vpr (65–79) peptide (QQLL*FIHFRIG*CQHS), which had been reported to exhibit good IN ST-inhibitory potency (IC_50_ = 14 µM) [22]. We found that the shortened sequence can exhibit low micromolar inhibitory potency in in vitro IN assays, with greater efficacy against the ST reaction relative to 3′-P reactions. The charge-state of the peptide terminal groups affected inhibitory potencies. Peptides having both termini free (**1a**; 3′-P IC_50_ = 18 ± 1 µM; ST IC_50_ = 1.3 ± 0.3 µM) or the *C*-terminus as a carboxamide and the amino terminus as its acetamide (**1c**; 3′-P IC_50_ = 24 ± 3 µM; ST IC_50_ = 4.7 ± 0.3 µM) were marginally more potent than the carboxamide peptide having a free amino terminus (**1b**; 3′-P IC_50_ = 32 ± 6 µM; ST IC_50_ = 7.3 ± 0.8 µM) (Table 1).

Next, to determine the relative contribution of each residue to overall inhibitory potency, we performed a sequential alanine scan on the peptides **1a**–**c**. This yielded three series of congeners **2a**–**c** through **8a**–**c**. Alanine substitution at all positions decreased IN inhibitory potencies relative to the parent peptides. Peptides containing a free amino terminus with a C-terminal carboxamide showed the lowest potency for a given alanine substitution (peptides **2b**–**8b**) (Table 1). The greatest loss of potencies occurred by substitution of the Arg (Vpr73), Phe (Vpr72) and Ile (Vpr70) residues. The Arg and Ile residues are of note, as they occupy the i and i + 4 positions on the same side of α-helix III that faces outward from the hydrophobic core comprised of α-helices I and II (Figure 1B). This could potentially make them accessible for interactions with other proteins. Interestingly, while replacing the amino-terminal Phe (Vpr69) residue with hydrophobic residues was generally well tolerated, replacement with Glu abrogated both 3′-P and ST-inhibitory potencies (IC_50_ values greater than 111 µM; data not shown). This may reflect formation of an intramolecular salt bridge arising between the anionic Glu side chain and the guanidinium moiety of the Arg (Vpr73) residue, which could interfere with the ability of this latter residue to engage in protein-protein interactions (PPIs) (refer to Figure 1B for side chain proximity).

### 2.2. Peptide ***1a*** Does Not Interfere with IN–DNA Binding or Induce Aberrant IN Multimerization 

**Figure 2:**
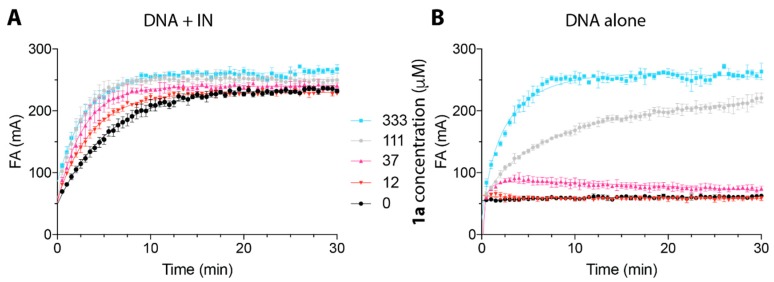
The binding properties of peptide **1a** to IN–DNA complex and DNA alone evaluated over time by fluorescence anisotropy or in the presence of IN (400 nM) preincubated with **1a** at the indicated concentrations (**A**) or in the absence of IN (**B**). Different concentrations of peptide **1a** are color-coded: 0 µM in black; 12 µM in red; 37 µM in pink; 111 µM in gray; 333 µM in blue.

To further our understanding of the mode of IN inhibition by Vpr-derived peptides, we evaluated the effect of **1a** on IN–DNA complex formation using fluorescence anisotropy. The results showed that preincubation of IN with **1a** did not block the IN–DNA interactions, but rather promoted IN–DNA complex formation (Figure 2A), as demonstrated by the increased fluorescence polarization upon treatment with increasing concentrations of **1a**. Consistent with this finding, the C-terminal Vpr peptides, including Vpr (65–79), have been shown to interact with IN [20]. We also investigated the binding of **1a** to the DNA substrate alone (Figure 2B) and found no detectable binding of **1a** to DNA at concentrations up to 37 µM, which were sufficient for inhibition of IN by **1a** [IC_50_ (3′-P) 17.6 µM, IC_50_ (ST) 1.3 µM, Table 1]. The increase in fluorescence at high concentrations of **1a**, [111 µM and 333 µM (Figure 2)], can potentially be associated with multimerization of **1a** [26]. Indeed, the Vpr Helix III motif residues within **1a**, (69–71 and 74) were shown to contribute to the Vpr dimerization interfaces via either parallel or antiparallel modes [26]. Because the increase of fluorescence polarization, which is indicative of **1a**–DNA binding, was only observed at concentrations of **1a** above 37 µM (Figure 2B), while this concentration did not disrupt IN–DNA complex formation, it is likely that **1a** multimers are not the IN-inhibiting species. These fluorescence experiments show that **1a** does not disrupt IN–DNA complexes and that it does not bind to DNA alone at concentrations relevant to IN inhibition. This suggests that **1a** exerts its inhibitory activity by binding to IN or IN–DNA complexes.

**Figure 3:**
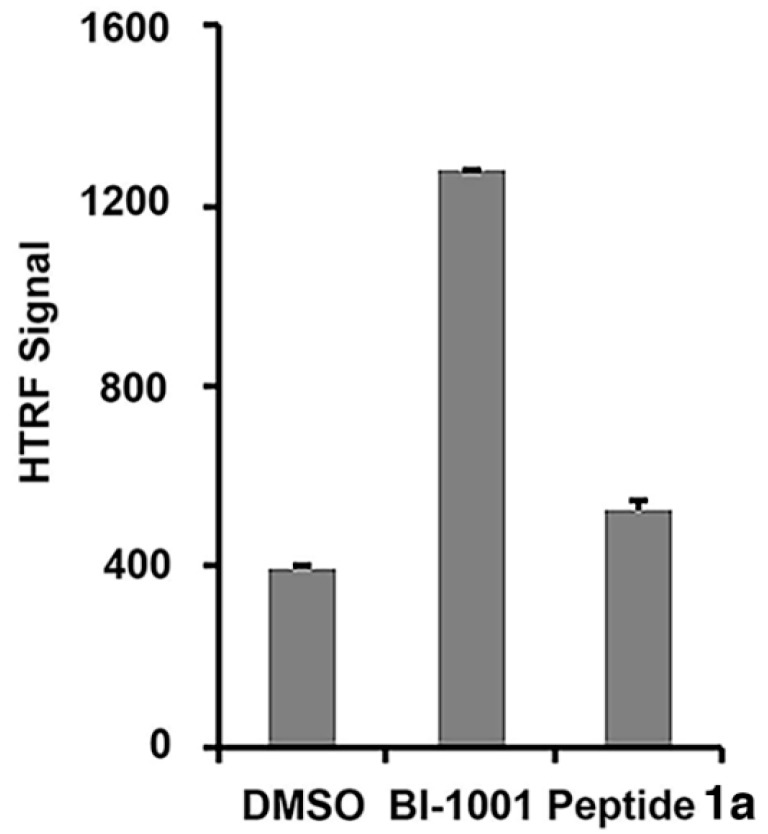
Effects of peptide **1a** on integrase multimerization. HTRF-based integrase multimerization signal recorded with a control ALLINI compound (**BI-1001**) [27] or 100 µM of peptide **1a**. Each data point represents the mean of three independent experiments.

Recently, allosteric HIV-1 IN inhibitors (ALLINIs) have been identified, which induce higher-order, aberrant IN multimerization in vitro and in virions, and inhibit proper virus particle maturation [27,28,29,30,31,32,33,34,35]. Using a homogeneous time-resolved fluorescence resonance energy transfer (HTRF) assay [36], we found that unlike a representative ALLINI (**BI-1001**), **1a** did not induce higher-order IN multimerization, suggesting an allosteric IN inhibition mechanism that differs from that used by ALLINIs (Figure 3) [27]. 

### 2.3. Development of a Vpr Peptide-Based Photoprobe

How Vpr-derived peptides interact with IN to inhibit its catalytic function is not known. However, identifying the region of IN engaged by the Vpr (69–75) peptides may clarify the processes involved. This could serve as a first step in developing novel allosteric inhibitors with a distinct mechanism from small molecule catalytic site-directed IN inhibitors. Photoaffinity crosslinking can be a highly useful means to identify ligand-binding regions. The benzophenone moiety is stable in ambient light, yet at wavelengths of light in the region 350–360 nm, photo-activation can occur, which can lead to covalent modification of C–H bonds, even under aqueous conditions [37,38,39]. We have previously incorporated benzophenones into coumarin-based IN inhibitors [40] and used this approach to identify their binding sites on HIV-1 IN through photo-crosslinking [41]. We have also applied this photophore to 2,4-dioxobutanoic acid-based IN inhibitors [42,43]. The benzoylphenylalanine (Bpa) variant of phenylalanine can be used to render peptides photo-reactive for the identification peptide-protein interactions [44].

It was unclear whether introducing a Bpa residue into the parent peptide **1a** could be done in a fashion that would not disrupt its binding to IN. Accordingly, beginning with the Vpr74 Ile residue, we conducted a positional scan that sequentially replaced each residue of the peptide with Bpa (peptides **9a**–**14a**). Substitution was notably well tolerated at nearly all positions examined. Relative to the parent peptide **1a**, replacement of the Vpr74 Ile residue with Bpa did not diminish inhibitory potency [**1a**, 3′-P IC_50_ = 18 µM, ST IC_50_ = 1 µM as compared to **9a**, 3′-P IC_50_ = 20 µM, ST IC_50_ = 4 µM (Table 1)]. Consistent with the results of the alanine scan, replacement of the Vpr73 Arg with Bpa gave the most dramatic loss of inhibitory potency as compared with substitutions of other Vpr residues (**10a**, 3′-P IC_50_ > 300 µM, ST IC_50_ ⋍ 50 µM). This further supports the hypothesis that this Arg residue may play a critical role in binding to IN. The second-most adverse impact on inhibitory potency occurred by substitution of the Vpr70 Ile residue (**13a**, 3′-P IC_50_ = 37 ± 5 µM, ST IC_50_ = 20 ± 3 µM). This also parallels results of the alanine scan, where substitution at this position had the next-to greatest effect (peptide **7a**). As pointed out above, the Vpr70 Ile and Vpr73 Arg residues occupy relative i and i + 4 positions within the α-helix III, which would place them on the same outward-facing side (Figure 1B). The best inhibitory potency was observed by replacing the Vpr69 Phe residue with Bpa [**14a**, 3′-P IC_50_ = 7.2 ± 1.2 µM, ST IC_50_ = 2.1 ± 0.3 µM) (Table 1)]. These values are approximately 10-fold lower than what was observed by replacing Phe with Ala (peptide **8a**).

**Figure 4:**
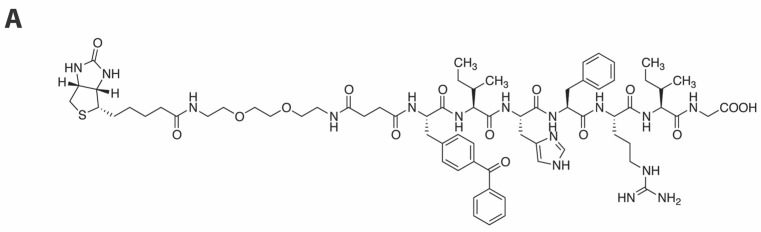

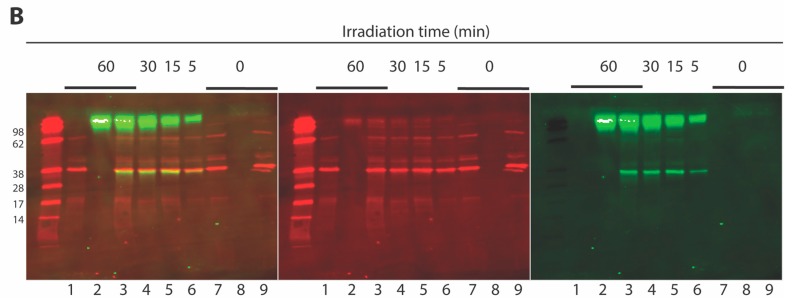
Integrase crosslinking studies. (**A**) Structure of photoprobe **15** showing inhibitory potencies in an in vitro IN assay. (**B**) Interaction of **15** with IN as evaluated by Western blotting after UV crosslinking (254 nM) and separation on Invitrogen Novex 4–20% Tris-Glycine gels (Invitrogen™ Novex™ SeeBlue™ Plus2 prestained protein standard is included in the outmost left lane): Lane 1, HIV-1 IN only (60 min UV); Lane 2, Peptide **15** only (60 min UV); Lane 3, IN and peptide **15** (60 min UV); Lane 4, 30 min UV; Lane 5, 15 min UV; Lane 6, 5 min UV; Lane 7, HIV-1 IN only; Lane 8, Peptide **15** only; Lane 9, IN and **15**. Gel stained with rabbit anti-IN CTD polyclonal (260–280)/anti-rabbit Licor, 680 nm (center, red), streptavidine-AlexaFluor 750 nm (right, green), merged image (left, merged). Molecular size markers are indicated at left (kDa).

Based on the above results, we selected peptide **14a** as a platform for further photoprobe construction. Because low crosslinking efficiency and high background noise can be problematic barriers to effective applications of photoaffinity ligands, biotin tags are often included as parts of the photoaffinity ligands to allow visualization and purification of covalent adducts [45,46,47]. Accordingly, we incorporated a biotin moiety by tethering it from the amino terminus using a hydrophilic linker and we found that inhibitory potency was retained [**15**, 3’-P IC_50_ = 32 ± 7 µM, ST IC_50_ = 2.3 ± 0.3 µM (Figure 4A)]. 

We incubated **15** with IN and subjected the mixture to UV irradiation followed by SDS-PAGE separation of protein products. The presence of IN was detected by Western blotting using an anti-IN C-terminal domain (CTD) polyclonal antibody (Figure 4B). The presence of **15** was revealed on the same Western blot membrane using streptavidin-coupled AlexaFluor 750. To facilitate the detection of crosslinking products by Western blot, relatively high concentrations of **15** (111 µM) and IN (3.2 µM) were used. The biotin-bound entities were visualized as green on the blot, whereas IN and some of the proteins from the prestained standard were colored red (Figure 4B). Upon UV irradiation of the preincubated mixture containing **15** and IN, a product was formed that contained biotin label and comigrated with IN on the gel (Figure 4B, Lanes 3–6). This indicated the presence of covalent adducts of **15** with IN. Such adducts are lacking when IN was irradiated in absence of **15** (Figure 4B, Lane 1) or when the mixture was not UV treated (Figure 4B, lane 9). Additionally, the crosslinked product (Figure 4B, Lanes 3–6) migrated at the same rate as the non-crosslinked IN (Figure 4B, Lane 7). This suggests that a monomeric IN subunit (molecular weight of 34.5 kDa) forms a single crosslink to **15** (molecular weight of 1449 Da), which indicates close to a 1:1 stoichiometry for **15**–IN interaction. In addition to the **15**–IN product, a high molecular weight product was formed, which also contained the biotin label (Figure 4B, Lanes 2–6, top bands). This product formed in the absence of IN (Figure 4B, Lane 2) and its accumulation was time-dependent (Figure 4B, Lanes 3–6). Formation of biotinylated high molecular weight product could be explained by polymerization of **15** upon irradiation. Potential multimerization of the Vpr-derived peptides would likely facilitate such polymerization. This corroborates the results of fluorescence anisotropy experiments, which demonstrated the binding of **1a** to DNA at high concentrations [111 µM and 333 µM (Figure 2)]. Despite polymerization of **15**, no reaction of polymeric product with IN could be detected as shown by UV irradiation of **15** in absence and in presence of IN (Figure 4B, Lanes 2 and 3, respectively). This further suggests that potential multimers of Vpr-derived peptide do not bind or inhibit IN. Although the multimerization of Vpr-derived peptide inhibitors of IN could not be entirely ruled out, our anisotropy and crosslinking results indicate that it is monomeric peptides (i.e., **1a** or **15**) that are responsible for IN inhibition.

## 3. Materials and Methods

### 3.1. General Synthetic

Preparative high-performance liquid chromatography (HPLC) was conducted using a Waters Prep LC4000 system (Milford, MA, USA) having photodiode array detection and a Phenomenex C_18_ column (00G-4436-P0-AX, 250 mm × 21.2 mm, 10 µm particle size, 110 Å pore Phenomenex, Torrance, CA, USA) at a flow rate of 10 mL/min. Binary solvent systems consisting of A = 0.1% aqueous trifluoroacetic acid (TFA) and B = 0.1% TFA in acetonitrile were employed with gradients, as indicated. Products were obtained as amorphous solids following lyophilization. Electrospray ionization-mass spectra (ESI-MS) and the purity of final peptides were determined with an Agilent LC/MS system (Santa Clara, CA, USA). Matrix-assisted laser desorption/ionization (MALDI) mass spectra were acquired on a Shimadzu Biotech Axima-CFR time-of-flight instrument (Columbia, MD, USA) using a-cyano-4-hydroxycinnamic acid as matrix. High-resolution mass spectra (HRMS) were acquired by LC/MS-ESI using an LTQ-Orbitrap-XL (ThermoFisher Scientific, Grand Island, NY, USA) at 30 K resolution.

### 3.2. Peptide Synthesis

Amino acid reagents, Fmoc-Gly-OH, Fmoc-Phe-OH, Fmoc-His(Trt)-OH, Fmoc-Ile-OH and Fmoc-Ala-OH were obtained from NOVA Biochem (MilliporeSigma, Burlington, MA, USA). Fmoc-Arg(Pbf)-OH, Fmoc-Ala-OH, Fmoc-4-benzoyl-l-phenylalanine [Fmoc-L-Bpa-OH], d-(+)-biotin and were obtained from Chem-Impex International, Inc (Wood Dale, IL, USA). The linker reagent for appending biotin to the amino terminus in peptide **15** [1-(9*H*-fluoren-9-yl)-3,14-dioxo-2,7,10-trioxa-4,13-diazaheptadecan-17-oic acid] was prepared from succinic anhydride and 2,2′-(ethylenedioxy)bis(ethylamine) according to literature procedures [48]. Fmoc-Gly-Wang resin and Rink amide MBHA (4-methylbenzhydrylamine) resin were obtained from Novabiochem.

Peptides were synthesized using standard Fmoc solid-phase peptide synthesis (SPPS) protocols using active ester coupling methodology in *N*-methylpyrrolidinone (NMP) using a CEM Liberty Microwave Peptide Synthesizer (CEM Corporation, Matthews, NC, USA). In summary, the protected amino acids or acid (5.0 eq. based on resin loading, 0.2 M in NMP), *O*-(benzotriazol-1-yl)-*N*,*N*,*N*′,*N*′-tetramethyluronium hexafluorophosphate (HBTU) (5.0 eq., 0.45 M in NMP) and *N*,*N*-diisopropylethylamine (DIEA) (5.0 eq., 2 M in NMP) were coupled using microwave irradiation (75 °C, 5 min, double couple). Fmoc deprotection was accomplished using 20% piperidine in NMP (with 0.1 M 1-hydroxybenzotriazole hydrate (HOBt). Where appropriate, *N*-terminal acetylation was achieved by manual coupling using 1-acetylimidazole (10% *w*/*v* in DMF). Final resins were washed sequentially with NMP, MeOH, CH_2_Cl_2_, and Et_2_O and dried under vacuum (overnight). Peptides were cleaved from the resin by treatment with a cocktail solution of TFA: triisopropylsilane (TIS): H_2_O (95: 2.5: 2.5; 5 mL, 4 h). The resin was removed by filtration and the peptide was precipitated in cold Et_2_O and the precipitate was centrifuged and washed with Et_2_O. The resulting white solid was dissolved in 50% aqueous acetonitrile (4 mL) and purified by reverse phase preparative HPLC using linear gradients as indicated below. Final peptides were characterized as indicated in the General Synthetic section using ESI-MS, MALDI-MS or HRMS. Data are presented below: 

*Peptide***1a***.* Linear gradient of 15% B to 30% B over 30 min; retention time = 25.9 min. ESI-MS calcd for C_44_H_65_N_12_O_8_ [MH^+^], 889.5; found, 889.3.

*Peptide***1b***.* Linear gradient of 15% B to 30% B over 30 min; retention time = 22.9 min. ESI-MS calcd for C_44_H_66_N_13_O_7_ [MH^+^], 888.5; found, 888.4. 

*Peptide***1c***.* Linear gradient of 20% B to 40% B over 30 min; retention time = 22.4 min. ESI-MS calcd for C_46_H_68_N_13_O_8_ [MH^+^], 930.5; found, 930.4. 

*Peptide***2a***.* Linear gradient of 15% B to 30% B over 30 min; retention time = 27.0 min. MALDI-MS calcd for C_45_H_67_N_12_O_8_ [MH^+^], 903.5; found, 903.7. 

*Peptide***2b***.* Linear gradient of 15% B to 30% B over 30 min; retention time = 24.2 min. ESI-MS calcd for C_45_H_68_N_13_O_7_ [MH^+^], 902.5; found, 902.4. 

*Peptide***2c***.* Linear gradient of 20% B to 40% B over 30 min; retention time = 22.3 min. ESI-MS calcd for C_47_H_70_N_13_O_8_ [MH^+^], 944.5; found, 944.4. 

*Peptide***3a***.* Linear gradient of 15% B to 25% B over 30 min; retention time = 20.7 min. ESI-MS calcd for C_41_H_59_N_12_O_8_ [MH^+^], 847.5; found, 847.4. 

*Peptide***3b***.* Linear gradient of 15% B to 25% B over 30 min; retention time = 18.2 min. MALDI-MS calcd for C_41_H_60_N_13_O_7_ [MH^+^], 846.5; found, 846.3. 

*Peptide***3c***.* Linear gradient of 20% B to 40% B over 30 min; retention time = 17.1 min. ESI-MS calcd for C_43_H_62_N_13_O_8_ [MH^+^], 888.5; found, 888.4. 

*Peptide***4a***.* Linear gradient of 15% B to 40% B over 30 min; retention time = 23.7 min. MALDI-MS calcd for C_41_H_58_N_9_O_8_ [MH^+^], 804.4; found, 804.6. 

*Peptide***4b***.* Linear gradient of 15% B to 25% B over 30 min; retention time = 28.5 min. MALDI-MS calcd for C_41_H_59_N_10_O_7_ [MH^+^], 803.5; found, 803.7. 

*Peptide***4c***.* Linear gradient of 20% B to 40% B over 30 min; retention time = 28.1 min. ESI-MS calcd for C_43_H_61_N_10_O_8_ [MH^+^], 845.5; found, 845.4. 

*Peptide***5a***.* Linear gradient of 10% B to 20% B over 30 min; retention time = 27.9 min. MALDI-MS calcd for C_38_H_61_N_12_O_8_ [MH^+^], 813.5; found, 813.5. 

*Peptide***5b***.* Linear gradient of 10% B to 20% B over 30 min; retention time = 23.4 min. MALDI-MS calcd for C_38_H_62_N_13_O_7_ [MH^+^], 812.5; found, 812.5. 

*Peptide***5c***.* Linear gradient of 20% B to 30% B over 30 min; retention time = 14.4 min. ESI-MS calcd for C_40_H_64_N_13_O_8_ [MH^+^], 854.5; found, 854.4. 

*Peptide***6a***.* Linear gradient of 15% B to 40% B over 30 min; retention time = 23.9 min. MALDI-MS calcd for C_41_H_63_N_10_O_8_ [MH^+^], 823.5; found, 823.7. 

*Peptide***6b***.* Linear gradient of 15% B to 30% B over 30 min; retention time = 29.1 min. ESI-MS calcd for C_41_H_64_N_11_O_7_ [MH^+^], 822.5; found, 822.3. 

*Peptide***6c***.* Linear gradient of 20% B to 40% B over 30 min; retention time = 28.8 min. ESI-MS calcd for C_43_H_66_N_11_O_8_ [MH^+^], 864.5; found, 864.4. 

*Peptide***7a***.* Linear gradient of 15% B to 25% B over 30 min; retention time = 25.9 min. MALDI-MS calcd for C_41_H_59_N_12_O_8_ [MH^+^], 847.5; found, 847.9.

*Peptide***7b***.* Linear gradient of 15% B to 25% B over 30 min; retention time = 21.9 min. ESI-MS calcd for C_41_H_60_N_13_O_7_ [MH^+^], 846.5; found, 846.4. 

*Peptide***7c***.* Linear gradient of 20% B to 40% B over 30 min; retention time = 18.4 min. ESI-MS calcd for C_43_H_62_N_13_O_8_ [MH^+^], 888.5; found, 888.4. 

*Peptide***8a***.* Linear gradient of 15% B to 25% B over 30 min; retention time = 25.9 min. ESI-MS calcd for C_38_H_61_N_12_O_8_ [MH^+^], 813.5; found, 813.3. 

*Peptide***8b***.* Linear gradient of 15% B to 25% B over 30 min; retention time = 21.4 min. ESI-MS calcd for C_38_H_62_N_13_O_7_ [MH^+^], 812.5; found, 812.8. 

*Peptide***8c***.* Linear gradient of 20% B to 30% B over 30 min; retention time = 17.9 min. ESI-MS calcd for C_40_H_64_N_13_O_8_ [MH^+^], 854.5; found, 854.4. 

*Peptide***9a***.* Linear gradient of 15% B to 50% B over 30 min; retention time = 22.0 min. ESI-MS *m*/*z*: 1027.3 (MH^+^). HRMS calcd for C_54_H_67_N_12_O_9_ [MH^+^], 1027.5148; found, 1027.5161.

*Peptide***10a**. Linear gradient of 20% B to 50% B over 30 min; retention time = 24.0 min. ESI-MS *m*/*z*: 984.4 (MH^+^). HRMS calcd for C_54_H_66_N_9_O_9_ [MH^+^], 984.4978; found, 984.4969. 

*Peptide***11a**. Linear gradient of 20% B to 35% B over 30 min; retention time = 24.9 min. ESI-MS *m*/*z*: 993.4 (MH^+^). HRMS calcd for C_51_H_69_N_12_O_9_ [MH^+^], 993.5305; found, 993.5310. 

*Peptide***12a**. Linear gradient of 20% B to 50% B over 30 min; retention time = 24.1 min. ESI-MS *m*/*z*: 1003.3 (MH^+^). HRMS calcd for C_54_H_71_N_10_O_9_ [MH^+^], 1003.5400; found, 1003.5402. 

*Peptide***13a**. Linear gradient of 20% B to 40% B over 30 min; retention time = 21.0 min. ESI-MS *m*/*z*: 1027.3 (MH^+^). HRMS calcd for C_54_H_67_N_12_O_9_ [MH^+^], 1027.5148; found, 1027.5168. 

*Peptide***14a**. Linear gradient of 20% B to 50% B over 30 min; retention time = 17.6 min. ESI-MS *m*/*z*: 993.4 (MH^+^). HRMS calcd for C_51_H_69_N_12_O_9_ [MH^+^], 993.5305; found, 993.5315. 

*Peptide***15**. Linear gradient of 20% B to 45% B over 30 min; retention time = 25.7 min. ESI-MS *m*/*z*: 1449.6 (MH^+^). HRMS calcd for C_71_H_101_N_18_O_15_S [MH^+^], 1449.7348; found, 1449.7358.

### 3.3. In Vitro Integrase Catalytic Assays

IN reactions were performed as previously described [49]. The reactions were carried by incubating a mixture of 20 nM [γ-^32^P]-labeled DNA and 400 nM IN in a buffer containing 50 mM morpholinepropanesulfonic acid (pH 7.2), 7.5 mM MgCl_2_, and 14 mM 2-mercaptoethanol in the absence or presence of peptide inhibitors in DMSO (vehicle) at eight three-fold dilutions ranging from 111 μM to 50 nM. Reactions were performed at 37 °C (2 h) and quenched by the addition of an equal volume of loading buffer (formamide containing 1% sodium dodecyl sulfate, 0.25% bromophenol blue, and xylene cyanol). Reaction products were separated in 16% polyacrylamide denaturing sequencing gels. Dried gels were visualized using a Typhoon 8600 instrument (GE Healthcare, Chicago, IL, USA). Densitometric analyses were performed using ImageQuant 5.1 software (GE Healthcare, Chicago, IL, USA). Data analyses (linear regression, 50% inhibitory concentration [IC_50_] determination, standard deviation [SD]) were performed from at least 3 independent determinations using Prism 5.0c software from GraphPad (La Jolla, CA, USA). To assay 3′-P inhibitory potency, reactions were carried out with [γ-^32^P]-labeled full-length oligonucleotide 21T (GTGTGGAAAATCTCTAGCAGT) annealed to the complementary oligonucleotide 21B (ACTGCTAGAGATTTTCCACAC). Formation of the shorter processed 19T products was monitored and quantified to assess enzyme performance and inhibitor potency. The ST reactions were carried out with the [γ-^32^P]-labeled pre-cleaved oligonucleotide 19T (GTGTGGAAAATCTCTAGCA) annealed to the complementary oligonucleotide 21B (ACTGCTAGAGATTTTCCACAC) and formation of integration products (longer and slower migrating than the starting 19T substrate) was monitored to assess enzyme performance and inhibitor potency.

### 3.4. Homogeneous Time-Resolved Fluorescence Resonance Energy Transfer (HTRF)-Based Protein-Protein Interaction Assays

WT HIV-1 IN recombinant proteins with His or FLAG tags [50] were expressed in *Escherichia coli* and purified as described previously [27]. The His_6_–tagged IN construct was purified by loading the cell lysate onto a Nickel-Sepharose column (GE Healthcare, Chicago, IL, USA) and eluting bound integrase with an imidazole gradient in a 50 mM HEPES (pH 7.5) buffer containing 1 M NaCl, 7.5 mM CHAPS, 2 mM beta-mercaptoethanol. Peak fractions were pooled, diluted and loaded onto a heparin column (GE Healthcare), and integrase was eluted with an increasing NaCl gradient in a 50 mM HEPES (pH 7.5) buffer containing 7.5 mM CHAPS and 2 mM beta-mercaptoethanol. The FLAG-tagged IN construct was purified by loading the ammonium sulfate precipitate of cell lysate onto a phenyl-Sepharose column (GE Healthcare) and eluting bound integrase with a decreasing ammonium sulfate gradient in a 50 mM HEPES (pH 7.5) buffer containing 200 mM NaCl, 7.5 mM CHAPS, 2 mM beta-mercaptoethanol. Peak fractions were pooled and loaded onto a heparin column (GE Healthcare), and integrase was eluted with an increasing NaCl gradient in a 50 mM HEPES (pH 7.5) buffer containing 7.5 mM CHAPS and 2 mM beta-mercaptoethanol. The HTRF-based IN multimerization assays were performed by mixing 6 × His-tagged and FLAG-tagged INs (each at 10 nM final concentration) in a 25 mM Tris (pH 7.4) buffer containing 150 mM NaCl, 2 mM MgCl_2_, 0.1% Nonidet P-40, 1 mg/mL BSA. Test compounds were then added to the mixture and incubated for 2.5 h at room temperature. 6.6 nM anti-His6-XL665 and 0.45 nM anti-FLAG-EuCryptate antibodies (Cisbio, Inc., Bedford, MA, USA) were then added to the reaction and incubated at room temperature for 3 h. The HTRF signal was recorded using a PerkinElmer EnSpire multimode plate reader [27].

### 3.5. DNA Binding Experiments

DNA binding was measured using a plate-based assay following fluorescence polarization of a fluorescent 21-mer duplex DNA substrate (21T/21B duplex oligonucleotide) with an AlexaFluor 488 modification at the 5′-end of the 21T oligonucleotide. Peptide or DMSO (control, without peptide) were incubated at room temperature for 5 min in the IN-activity buffer in the absence or the presence of IN (400 nM). After addition of the DNA (10 nM), fluorescence anisotropy was measured every 30 s for 30 min using an Envision plate reader (Perkin Elmer, Waltham, MA, USA).

### 3.6. Crosslinking Experiments

Peptide **15** (111 µM), containing a photo-reactive Bpa group and a biotin linked to its amino terminus, was incubated for 2 h at 37 °C in the absence or the presence of IN (3.2 µM) before UV irradiation (5 min at 254 nM). After adding 2 × 10 µL of loading buffer, samples were heat-denatured at 95 °C (5 min). Products were separated on 16% SDS-PAGE and transferred to a PVDF membrane using an iBlot system (Invitrogen, Carlsbad, CA, USA). IN was revealed by Western blot using a primary rabbit polyclonal antibody directed against the IN CTD (AIDS Reagent program, cat. No. 758) and an anti-rabbit secondary antibody from Li-COR (680 nm), while the biotinylated peptide was revealed using streptavidin coupled to AlexaFluor 750. Reading was performed using an Odyssey^®^ Infrared Imaging System (LI-COR Biotechnology, Lincoln, NE, USA).

## 4. Conclusions

Vpr-derived peptides have previously been shown to inhibit IN function in in vitro assays. Identification of the sites where these peptides interact with IN could potentially facilitate the development of a new class of IN inhibitors. Starting with the Vpr (69–75) sequence found in the α-helix III, we developed peptide **1a**, which inhibits IN in vitro at low micromolar concentrations by a mechanism that does not appear to involve disrupting IN–DNA interactions. Furthermore, unlike ALLINIs, peptide **1a** fails to promote aberrant IN multimerization. We have further identified the outward-facing i and i + 4 α-helix III residues Vpr73 Arg and Vpr70 Ile as being particularly important for IN inhibitory potency, which may suggest that these residues interact with IN. Collectively, our findings indicate that the mode of action of peptide **1a** is distinct from both clinically used INSTIs and investigational ALLINIs.

To define the interactions of Vpr-derived peptides with IN, we prepared **15** as a biotinylated photoprobe variant of peptide **1a** and demonstrated its ability to covalently bind to IN. It should be noted that the same helix III motif of Vpr, which gives rise to the peptide IN inhibitors, is also involved in interactions of Vpr with the human Vpr-binding protein DCAF1 [51]. This further supports the potential of Vpr-derived biotinylated probes, such as **15**, to investigate the specificity of Vpr-based IN inhibitors and their role and potential binding partners. Peptide **15** may facilitate studies directed at the development of Vpr-based allosteric inhibitors.

## Figures and Tables

**Table 1:** Structures of peptides and 3′-P and ST-inhibitory potencies in in vitro IN assays.

No.	SEQUENCE *^i^*	3’-P IC_50_ (µM)	ST IC_50_ (µM)
**1a**	FIHFRIG	17.6 ± 1.2	1.3 ± 0.3
**1b**	FIHFRIG-amide	31.5 ± 6	7.3 ± 0.8
**1c**	Ac-FIHFRIG-amide	23.5 ± 3	4.7 ± 0.3
**2a**	FIHFRI**A**	>111	34.7 ± 2.3
**2b**	FIHFRI**A**-amide	>111	60.6 ± 8
**2c**	Ac-FIHFRI**A**-amide	74.5 ± 7	30.4 ± 0.7
**3a**	FIHFR**A**G	66.5 ± 7	22.4 ± 1.7
**3b**	FIHFR**A**G-amide	> 111	>111
**3c**	Ac-FIHFR**A**G-amide	208 ± 12	28 ± 4
**4a**	FIHF**A**IG	>111	>111
**4b**	FIHF**A**IG-amide	>111	>111
**4c**	Ac-FIHF**A**IG-amide	>333	81 ± 9
**5a**	FIH**A**RIG	>111	46.6 ± 6.5
**5b**	FIH**A**RIG-amide	>111	>111
**5c**	Ac-FIH**A**RIG-amide	>333	117 ± 10
**6a**	FI**A**FRIG	17.4 ± 2.0	6.7 ± 1.0
**6b**	FI**A**FRIG-amide	95.4 ± 5	35.0 ± 3.2
**6c**	Ac-FI**A**FRIG-amide	94 ± 13	3.5 ± 0.3
**7a**	F**A**HFRIG	>111	73.5 ± 6.0
**7b**	F**A**HFRIG-amide	>111	>111
**7c**	Ac-F**A**HFRIG-amide	>333	127 ± 14
**8a**	**A**IHFRIG	86.3 ± 5.0	22.7 ± 1.4
**8b**	**A**IHFRIG-amide	>111	>111
**8c**	Ac-**A**IHFRIG-amide	>333	46 ± 8
**9a**	FIHFR**B**G	20 ± 3	4.3 ± 1.3
**10a**	FIHF**B**IG	>333	48 ± 8
**11a**	FIH**B**RIG	34 ± 6	4.2 ± 0.7
**12a**	FI**B**FRIG	6.5 ± 0.9	2.2 ± 0.6
**13a**	F**B**HFRIG	37 ± 5	20 ± 3
**14a**	**B**IHFRIG	7.2 ± 1.2	2.1 ± 0.3
**15**	Biotin-linker-**B**IHFRIG	32 ± 7	2.3 ± 0.3

*^i^* “A” represents “Ala, Alanine”, “B” represents “Bpa, benzoylphenylalanine”.
